# Rapid and Reproducible Differentiation of Hematopoietic and T Cell Progenitors From Pluripotent Stem Cells

**DOI:** 10.3389/fcell.2020.577464

**Published:** 2020-10-20

**Authors:** Léa Flippe, Anne Gaignerie, Céline Sérazin, Olivier Baron, Xavier Saulquin, Maria Themeli, Carole Guillonneau, Laurent David

**Affiliations:** ^1^Université de Nantes, Inserm, Centre de Recherche en Transplantation et Immunologie, UMR 1064, ITUN, Nantes, France; ^2^LabEx IGO “Immunotherapy, Graft, Oncology”, Nantes, France; ^3^Université de Nantes, CHU Nantes, Inserm, CNRS, SFR Santé, FED 4203, Inserm UMS 016, CNRS UMS 3556, Nantes, France; ^4^Department of Pediatric Cardiac Surgery, University Hospital of Nantes, Nantes, France; ^5^Université de Nantes, CNRS, Inserm, CRCINA, Nantes, France; ^6^Department of Hematology, Cancer Center Amsterdam, Amsterdam University Medical Center (UMC), Amsterdam, Netherlands

**Keywords:** hiPSC, hESC, hematopoietic progenitor, T-cell progenitor, hematopoietic differentiation

## Abstract

Cell therapy using T cells has revolutionized medical care in recent years but limitations are associated with the difficulty of genome editing of the cells, the production of a sufficient number of cells and standardization of the product. Human pluripotent stem cells (hPSCs) can self-renew and differentiate into T cells to provide a standardized homogenous product of defined origin in indefinite quantity, therefore they are of great potential to alleviate limitations of therapeutic T cell production. The differentiation of hPSCs takes place in two steps: first the induction of hematopoietic stem/progenitor cells (HSPCs), then the induction of lymphopoiesis by Notch signaling. However, the differentiation of T cells from hPSCs can be difficult and lack reproducibility. One parameter that needs to be better assessed is the potential of DLL1 vs. DLL4 ligands of the Notch pathway to induce T cells. In addition, culture of hPSCs is labor-intensive and not compatible with GMP production, especially when they are cultured on feeder cells. Thus, the definition of a robust GMP-compatible differentiation protocol from hPSCs cultured in feeder-free conditions would increase the accessibility to off-the-shelf hematopoietic and T cell progenitors derived from hPSCs. In this article, we describe an efficient, rapid and reproducible protocol for the generation of hematopoietic and T cell progenitors in two steps: (1) generation of HSPCs from embryoid bodies (EB) in serum free medium and GMP-compatible feeder-free systems, (2) directed differentiation of hPSC-derived HSPCs into T-cell progenitors in the presence of bone marrow stromal cells expressing Notch-ligands OP9-DLL1 vs. OP9-DLL4.

## Introduction

Immunotherapy offers novel opportunities for unmet therapeutic needs. The ability to use T cells to directly target tumors has proven extremely useful for treating hematological cancer, such as B cell lymphoma and multiple myeloma, and solid cancers, such as metastatic melanoma and carcinoma (Fournier et al., 2017). T cells with regulatory properties could also be used in the context of immunoregulation, for example for grafted patients. In that case, immune cells would be injected to induce tolerance for the graft, while preserving the immune response of grafted patients (Bézie et al., 2017, 2019; Flippe et al., 2019). Despite great potential, some limitations impair the development of T cell therapy. One limitation is that during the necessary amplification of T cells for treatment, T cells ability exhausts. This is reinforced in conditions where the patient’s immune system is weakened by the treatment or the disease, thus precluding autologous T cell expansion. Another limitation is that T cells are difficult to edit, limiting their use with engineered antigen receptors. Altogether, T cell therapy is powerful but limited by the intrinsic nature of specialized cells (Nianias and Themeli, 2019).

A way to generate large numbers of T cells would be to amplify stem or progenitor cells and subsequently differentiate them into T cells. Human pluripotent stem cells (hPSCs) can self-renew and differentiate into any cell of the body, including hematopoietic cells. hPSCs are either human embryonic stem cells (hESCs) derived from blastocysts, or human induced pluripotent stem cells (hiPSCs) reprogramed from somatic cells (Thomson et al., 1998; Takahashi and Yamanaka, 2006; Takahashi et al., 2007). Since their derivation in 1998, hESCs have become a useful model for the study of human development or analysis of lineage and differentiation *in vitro*. In 2006, induced pluripotent stem cells (iPSCs) were obtained by reprograming mouse and human adult somatic cells using transduction of four factors essential for pluripotency. hiPSCs can be generated from patients with different diseases, therefore they can be used to obtain cells that carry genetic specificities and thus study the impact of this disease on development. Related to T lymphocytes, T cell clones with a desired antigenic specificity can be reprogramed into iPSC, which can be differentiated into an unlimited number of T lymphocytes preserving their given antigenic specificity (Kawamoto et al., 2018). Altogether, hiPSCs and hESCs are valuable tools for studying human development, disease modeling, drug screening and could provide an unlimited number of cells for regenerative medicine and cell therapy.

Some protocols for T cell differentiation are available (Timmermans et al., 2009; Mohtashami et al., 2010; Kennedy et al., 2012; Nishimura et al., 2013; Themeli et al., 2013; Sturgeon et al., 2014; Zhu et al., 2015). They rely on two steps: first, the differentiation of HSPCs from manque petit hPSCs, second, the differentiation of those progenitors into T cells. These protocols necessitate the use of hPSCs cultured on feeders, which limits the amount of T cells that can be subsequently generated. Moreover, these protocols are not standardized and the yield remains variable from one cell line to another. Therefore, better defined protocols are still needed.

There are several methods to differentiate hPSCs into hematopoietic progenitors: in co-culture with OP9 stromal cells (Holmes and Zúñiga-Pflücker, 2009; Ditadi and Sturgeon, 2016), in 2D (Salvagiotto et al., 2011; Slukvin, 2013) and 3D cultures through the formation of embryoid bodies (EBs) (Cerdan et al., 2007; Ng et al., 2008). These methods generally rely on induction of mesoderm through BMP signaling (Beddington and Robertson, 1999; Langdon and Mullins, 2011), followed by hemangiogenic induction by VEGF, SCF, FLT3l, IL3, and FGF2 (Ackermann et al., 2015) and after roughly a week of culture, the cells express CD34, a major marker of hematopoietic progenitors. The objective of this first step of differentiation is thus to obtain hematopoietic progenitors with a specific surface marker signature: CD43^+^, CD45^+^, KDR^–^.

Differentiation protocols aim to recapitulate the development of lymphocytes. Hence, the induction of lymphopoiesis relies on stimulation by SCF, FLT3l, IL7, and Notch signaling (Moore and Zlotnik, 1997; Radtke et al., 1999, 2004; Politikos et al., 2015). Differentiation effectiveness can be assessed by surface markers. The first expected event is that cells stop expressing CD34. The lymphopoiesis can then be followed by the subsequent expression of CD7, CD5 and finally the main markers CD4 and CD8.

In this study, we assessed parameters that would influence the robustness of hematopoietic and T-lineage cells differentiation from hPSCs. We used a protocol in two steps: (1) induction of hematopoietic differentiation through embryoid body (EB) formation in serum free medium and under two different pre-differentiation culturing conditions (feeder or feeder-free); (2) directed differentiation of hPSC-derived multipotent HSPCs into T-lineage cells by co-culture with OP9-DLL1 or OP9-DLL4 bone marrow stromal cells.

We efficiently generated human CD34^+^CD45^+^CD43^±^KDR^–^ hHSPCs from hPSCs maintained in two different types of culture, a co-culture with mitotically-inactivated mouse embryonic fibroblast (MEF) (i.e., feeder) and a culture on Matrigel-coated plates (i.e., feeder-free). The use of feeder-free cultured hPSCs resulted in efficient hHSPC induction, with yields similar to feeder-cultured hPSCs. We subsequently differentiated hHSPCs into CD7^+^ T lineage progenitors efficiently, with no noticeable difference between OP9-DLL1 or OP9-DLL4 co-culture. Within the T lineage progenitor population, we detected CD4^+^CD8^+^, CD4^+^CD8^–^ and CD4^–^ CD8^+^ populations.

## Materials and Equipments

### Cell Lines

- hES cells: hESC lines H1 (WA01, lot WB0111, male, *n* = 1) and H9 (WA09, lot WB0090, female, *n* = 21) were obtained from the WiCell Research Institute, under authorization RE13-004 from the French embryo research oversight committee, Agence de la Biomédecine.
- Transgene-free hiPS cells: 4 cell lines from male T-cell reprograming, T04.01A (*n* = 20), T04.01B (*n* = 6), T05.003 (*n* = 5) and T05.006 (*n* = 8) (Flippe et al., 2019). Two cell lines from male fibroblasts i.e., Lon71.019 (*n* = 9) (Gaignerie et al., 2018) and BJ1.B1 (*n* = 1) (Kilens et al., 2018). Four cell lines from female fibroblast reprograming i.e., Lon80.B2 (*n* = 1) (Gaignerie et al., 2018), MiPS203.B3 (*n* = 1), MiPS209.003 (*n* = 5) and MiPS220.003 (*n* = 5) (Kilens et al., 2018).

All cell lines were generated in the iPSC core facility of the University of Nantes.

- Mouse embryonic fibroblasts (MEFs) mitotically-inactivated (produced in house from SWISS/SWISS mice).
- OP9-DLL1 or DLL4 mouse bone marrow stromal cell lines expressing the delta-like ligand of the Notch pathway (kindly provided by Dr. Juan-Carlos Zuniga-Pflucker, University of Toronto).

### Reagents

- Matrigel (Corning Cat# 354277).
- Gelatin solution, type B (Sigma Cat# G1393).
- 0.5% Trypsin-EDTA (Life Technologies Cat# 25300-054).
- Dispase, 5 U/ml (StemCell Technologies Cat# 07913).
- mTeSR1 (StemCell Technologies Cat# 85850).
- DMEM F-12 (Life Technologies Cat# 31330-038.
- DMEM, high glucose, GlutaMAX Supplement (Life Technologies Cat# 61965026).
- α-MEM (Life Technologies Cat# 22561-021).
- Stempro-PRO-34 SFM + Supplement (Life Technologies Cat# 10639-011).
- KnockOut^TM^ Serum Replacement (KSR, Life Technologies Cat# A31815-02).
- Fetal Bovine Serum (Hyclone Cat# SV30160.03).
- GlutaMAX Supplement (Life Technologies Cat# 35050-038).
- Penicillin-Streptomycin, 10,000 U/ml (Life Technologies Cat# 15140-122).
- Non-essential amino acids (NEAA; Life Technologies Cat# 11140-035).
- 2-Mercaptoethanol, 50 mM (Life Technologies Cat# 31350-010).
- L-Ascorbic acid (Sigma Cat# A7506).
- Y-27632 dihydrochloride (RockI; Axon Cat# 1683).
- Sodium Pyruvate (Life Technologies Cat# 11360-039).
- Phosphate Buffered Saline (PBS; Life Technologies Cat#14190144).
- Recombinant cytokines – see Table 1
- Anti-human antibodies for flow cytometry – see Table 2

**TABLE 1:** Recombinant cytokines used for hPSCs culture and differentiation.

**Cytokines**	**Supplier**	**Catalog #**
FGF2	PeproTech	100-18B
BMP4	R&D Systems	314-BP
VEGF121	PeproTech	100-20A
SCF	PeproTech	300-07
FLT3l	PeproTech	300-19
IL3	PeproTech	200-03
IL7	PeproTech	200-07

**TABLE 2:** Antibodies used for analysis of differentiation cultures.

**Antigens**	**Clones**	**Conjugate**	**Supplier**	**Catalog #**	**Dilution**
CD34	581	PE-Cy7	BD Biosciences	560710	1/30
CD43	1G10	APC	BD Biosciences	560198	1/30
CD45	2D1	APC-Cy7	BD Biosciences	557833	1/40
KDR	89106	PE	BD Biosciences	560494	1/30
CD7	M-T701	PECF594	BD Biosciences	562541	1/25
CD5	UCHT2	BV711	BD Biosciences	563170	1/25
CD8	SK1	BV605	BD Biosciences	564116	1/25
CD4	RPA-T4	FITC	BD Biosciences	555346	1/25
CD8b	2ST8.5H7	PE	BD Biosciences	641057	1/25
CD56	B159	FITC	BD Biosciences	562794	1/30

### Equipments

- 6-Well tissue culture plates (Falcon).
- 6-Well low-adherence tissue culture plates (Corning).
- 6-cm culture dishes (Falcon).
- 5, 15, and 50 ml sterile centrifuge tubes (Falcon).
- 2, 5, 10, 25, and 50 ml sterile serological pipets (Falcon).
- 0.22 μm filtration system.
- Cell culture centrifuge.
- 5% CO_2_ incubator set at 37°C.
- Biosafety hood.
- Water bath set at 37°C.
- 4°C refrigerator.
- −20°C freezer.
- −150°C freezer.
- FACS CANTO and FACS LSRII or equivalent.
- FlowJo software.

### Reagents Preparation

#### Matrigel Solution

Thaw matrigel at 4°C overnight. Place the bottle of Matrigel (1 mg/ml) and tubes for aliquots on ice and aliquot matrigel 250 –500 μL in cold tubes with cold tips. Store at −80°C.

For use, thaw the 250–500 μl aliquot at 4°C overnight. Place the aliquot and a 50 ml tube on ice and resuspend the matrigel at a final concentration of 0.01 mg/ml in cold DMEM-F12.

#### 0.1% Gelatin Solution

Prepare a solution of 0.1% gelatin in PBS 1X and filter it at 0.22 μm. Store at 4°C.

#### MEF Culture Medium

Supplement DMEM with FBS to a final concentration of 10%, add NEAA and sodium pyruvate to a final concentration of 1%. Store at 4°C.

#### hPSC mTeSR1 Medium (Feeder-Free Culture)

Prepare as per manufacturer’s protocol. Aliquot in 50 ml tubes, pipetting no more than 40 ml per tube to allow for volume expansion upon freezing. Store at −20°C for storage or store at 4°C for immediate use.

#### hPSC-KSR Medium (Feeder Culture)

Supplement DMEM-F12 with KSR serum to a final concentration of 20%, add NEAA and Glutamax to a final concentration of 1% and add 50 μg/ml of 2-Mercaptoethanol. Divide into 50 ml aliquots and store at −20°C or store at 4°C for immediate use. Immediately prior to use, supplement with FGF2 to a final concentration of 10 ng/ml.

#### EB Medium

Prepare Stempro-PRO-34 SFM as per manufacturer’s protocol and add 2 mM of GlutaMAX Supplement. Divide into 40 ml aliquots and store at −20°C.

Prepare EB medium extemporaneously: thaw an aliquot of Stempro-PRO-34-SFM and add 50 μg/ml of 2-Mercaptoethanol, 1% of NEAA and 50 μg/ml L-Ascorbic acid.

#### OP9 Medium

Supplement α-MEM with 20% FBS and 2 mM GlutaMAX Supplement. Store at 4°C.

#### OP9 Differentiation Medium

Supplement OP9 medium with 50 μg/ml 2-Mercaptoethanol, 1% of NEAA, 50 μg/ml L-Ascorbic acid and 1% of Penicillin-Streptomycin. Store at 4°C.

## hESCs/hiPSCs Culture

### Feeder-Free Condition

(1) At least 4 h (we recommend overnight) before thawing hPSCs, coat 6-well plates with Matrigel and incubate at 37°C.
(2) Add 5 ml of mTeSR1 to 15 ml tube.
(3) Thaw a cryovial of hPSCs (frozen in CryoStore CS10 according to the manufacturer’s protocol, Stemcell Technologies) in a 37°C water bath.
(4) Add 1 ml of mTeSR1 dropwise to the cryovial and transfer the cell solution into the 15 ml tube containing 5 ml of mTeSR1.
(5) Centrifuge at 160 g 5 min.
(6) Gently resuspend cells in 1.5 ml of mTeSR1 + 10 μg/ml Y27632. Transfer 1/3rd (500 μl) of the cells in a well containing 1 ml of mTeSR1 + 10 μg/ml Y27632, the other 2/3rd (1 ml) in a well containing 500 μl of mTeSR1 + 10 μg/ml Y27632.
(7) Change mTeSR1 daily until hPSC are 70–80% confluent.
(8) At least 4 h (we recommend overnight) before passing hPSCs, coat new 6-well plates with Matrigel and incubate at 37°C.
(9) Remove mTeSR1, wash with 1 ml of PBS 1X and add 1 ml of StemMACS passaging solution XF for 3 min at room temperature. Aspirate and add 1 ml of mTeSR1. Scrape cells back and forth in two directions in the well with 200 μl tips.

NB: Be careful with the timing of incubation with the passaging solution. You might have to test multiple timings depending on your tissue culture practices. This step generates clumps of hPSCs that should be around 200 μm in size.

(10) Take the chosen volume of cell solution and add into a new well containing 1.5 ml of mTeSR1. Be careful to properly homogenize the cells and place at 37 °C under 5% CO_2_.

NB: The passaging ratio of hPSCs can vary from line-to-line, is very dependent of the lab and the manipulator. Usually for us, hPSCs are passed every 5–6 days at a ratio of 1/10.

### Feeder Condition

(1) At least 30 min before thawing mitotically-inactivated MEFs (Supplementary Material), coat 6 cm culture dishes with 1.5 ml of gelatin.
(2) Thaw mitotically-inactivated MEFs (frozen in 80% FBS and 80% Dimethyl Sulfoxide: DMSO) in a 37°C water bath. Add 1 ml of MEF medium dropwise to the vial and add the cell solution into the 50 ml tube containing 5 ml.
(3) Centrifuge at 200 g for 5 min.
(4) Resuspend in 10 ml of MEF medium and count the cells. Aspirate the gelatin and plate 2.5 ml of MEF solution into each 6-cm culture dish for a final density of 4.2 × 10^5^ cells per dish. Be careful to homogenize the cells well and place at 37 °C under 5% CO_2_ overnight.
(5) The following day, thaw a cryovial of hPSCs in a 37°C water bath.
(6) Add 5 ml of KSR medium in a 15 ml tube. Add 1 ml of KSR medium dropwise to the vial and add the cell solution into the 15 ml tube containing 5 ml of KSR medium.
(7) Centrifuge at 160 g for 5 min.
(8) Gently resuspend in 3 ml of KSR medium + 10 ng/ml FGF2 + 20 μg/ml Y27632. Remove the gelatin from the dish and add cell solution in the first dish at a ratio of 1/3 and a final volume of 3 ml. The second dish is complete at a ratio of 2/3 and a final volume of 3 ml. Be careful to homogenize the cells well and place at 37°C under 5% CO_2_.
(9) Every day, replace the KSR medium + 10 ng/ml FGF2 until hPSC are 70–80% confluent.
(10) A day before the passage, MEFs are prepared as described in the section “Materials and Equipments, hESCs/hiPSCs Culture, Feeder Condition (1-3).”
(11) The day of passage, cut colonies in a grid pattern (about 200 μm) with a needle under a magnifying glass. Collect the pieces of colonies with a pipette of 200 μl and transfer about 20 colonies in a new 6-cm dish containing already 2.5 ml of KSR medium + 10 ng/ml FGF2. Be careful to homogenize the cells well and place at 37 °C under 5% CO_2_.

NB: The passaging ratio of hPSCs can vary from line-to-line, it is very dependent of the lab and the manipulator. Usually for us, hPSCs are passed every 6–7 days at a ratio of 1/4.

### Embryoid Bodies Formation

#### Feeder-Free Condition

(1) 5–6 days before starting EB formation, pass the hPSCs as described in the section “Materials and Equipments, hESCs/hiPSCs Culture, Feeder-Free Condition (8-10).”
(2) Day – 1: Start the process when the cells are 70% confluent. Remove mTeSR1, wash with 1 ml of PBS and add 1 ml of StemMACS passaging solution XF for 3 min at room temperature. Very gently remove and wash with 1 ml of α-MEM. Add 1 ml of mTeSR1 and scrape cells back and forth in two directions of the well with 200 μL tips.

NB: For this step the generated clumps of hPSCs should be bigger than for a simple passage around 300–350 μm in size.

(3) Very carefully transfer the cell clumps with a 5 ml pipette into a low binding plate containing already 1 ml of mTeSR1 + 20 μg/ml Y27632.

NB: Generally, for each 6-well matrigel-coated plate containing hPSCs, one 6-well low binding plate for differentiation can be used.

(4) Homogenize the cells and place at 37 °C under 5% CO_2_ for no more than 18 h.

NB: Be careful, the Y27632 exposure time is really important for this step. If the exposure time exceeds 18 h, the embryoid bodies tend to disintegrate.

(5) Day 0: Collect EBs very gently with a 5 ml pipette into a 15 ml tube (1 tube for 1 well). Put 1 ml of “Day 0” EB medium (Table 3) into the well to prevent it from drying. Wait a few minutes to let the EBs sediment at the bottom of the tube and remove the supernatant. Gently resuspend the EBs in the “Day 0” EB medium and very carefully transfer into the well already containing 1 ml of medium. Place the plate at 37 °C under 5% CO_2_.
(6) NB: 24 h after the addition of BMP4 media, up to 20% of EBs collapsed due to the switch of medium from mTeSR1 to EB medium. This collapse might be evaluated by observation with phase contrast microscopes.
(7) Day 1: repeat the step (5) with “Day 1” EBs medium (Table 3).
(8) Idem for days 3, 5, and 7 (Table 3).
(9) Keep the cells until day 9 to have Hematopoietic Stem Cells.

**TABLE 3:** EB complete medium formulation for hematopoietic cell induction.

**EBs Medium**	**BMP4 (ng/ml)**	**FGF2 (ng/ml)**	**VEGF (ng/ml)**	**SCF (ng/ml)**	**FLT3l (ng/ml)**	**IL3 (ng/ml)**
Day 0	30	–	–	–	–	–
Day 1	30	5	–	–	–	–
Day 3	–	5	20	100	20	20
Day 5	–	5	20	100	20	20
Day 7	–		20	100	20	20

#### Feeder Condition

(1) Pass the hPSCs as described in the section “Materials and Equipments, hESCs/hiPSCs Culture, Feeder Condition (9-11)” 6–7 days before starting EB formation.
(2) Day 0: start the process when the cells are 70% confluent. Remove KSR medium, wash with 1 ml of PBS 1X and add 1.5 ml of dispase (1U/ml) for 10 min at 37°C. With a 5 ml pipette, add 2 ml of DMEM-F12 and flush the colonies no more than 3 times. Collect the clumps and put them into a 15 ml tube (1 tube for 1 dish). Proceed identically until there are no more colonies in the dish.
(3) Centrifuge at 160 g for 5 min.
(4) Resuspend the clumps with 5 ml DMEM-F12 and centrifuge again at 160 g for 5 min.
(5) Resuspend the pellet of clumps very gently into 2 ml of “Day 0” EB medium and transfer the cells very carefully with a 5 ml pipette into a low binding plate. Homogenize the cells and place at 37°C under 5% CO_2_.

NB: Typically, colonies from two 6-cm dishes go into one well of a 6-well low binding plate in 2 ml of EB medium.

(6) Day 1: Collect EBs very gently with a 5 ml pipette in a 15 ml tube (1 tube for 1 well). Put 1 ml of “Day 1” EBs medium (Table 3) into the well to prevent it from drying. Wait a few minutes to let the EBs sediment at the bottom of the tube and remove the supernatant. Gently resuspend the EBs in the “Day 1” EB medium and very carefully transfer into the well already containing 1 ml of medium. Place the plate at 37°C under 5% CO_2_.
(7) Day 3: repeat the step (6) with “Day 3” EBs medium (Table 3).
(8) Same for day 5 and day 7 (Table 3).
(9) Keep the cells until day 9 to obtain Hematopoietic Stem Cells.

### Hematopoietic Progenitor Analysis

(1) At days 7 and 9, collect one 6-well low binding plate of EBs with a 5 ml pipette and transfer them into a 15 ml tube. Rinse the well with 2 ml of PBS 1X.

NB: One 6-well low binding plate in one 15 ml tube.

(2) Centrifuge for 5 min at 450 g.
(3) Remove the supernatant and resuspend in 1 ml of Accutase. Incubate 15 min at 37°C.
(4) Pipette up and down with 1 ml tips to break the EBs.

NB 1: This step requires about 5 min per tube to sufficiently break the EBs.

NB 2: If the EBs are not yet broken, do an extra 5–10 min at 37°C.

(5) Add 5 ml of PBS 1X and centrifuge 7 min at 450 g.
(6) Resuspend the cells in 1 ml of FACS buffer and pass them through a 60 μm cell filter.
(7) Wash the cells with FACS buffer by centrifugation at 450 g for 7 min. Keep 1/3 of the cells from each condition and use them for the unstained and isotype control conditions. Incubate the cells with anti-hCD34, anti-hCD43, anti-hCD45 and anti-hKDR antibodies in FACS buffer for 30 min at 4°C in the dark (Table 2).
(8) Wash the cells twice with FACS buffer and resuspend them in PBS 1X + DAPI.
(9) Analyze the cells using flow cytometry.

## Lymphocyte Differentiation

### OP9-DLL1 or OP9-DLL4 Culture

#### Thawing

(1) At least 30 min before thawing OP9-DLL1 or DLL4 cells, coat two 10-cm dishes with 5 ml of 0.1% gelatin.
(2) Add 10 ml of OP9 medium to a 50 ml tube.
(3) Thaw a cryovial of OP9-DLL1 or OP9-DLL4 (frozen in 80% FBS and 20% DMSO) cells quickly in a 37°C water bath.
(4) Add 1 ml of OP9 medium dropwise to the vial and add the cell solution to the 50 ml tube containing 10 ml of OP9 medium.
(5) Centrifuge at 200 g for 5 min.
(6) Resuspend the cells in 3 ml of OP9 medium.
(7) Aspirate the gelatin. Add in one 10-cm dish, 8 ml of OP9 medium, and 9 ml of OP9 medium in the other.
(8) Transfer the cell solution into the two 10-cm dishes to a final volume of 12 ml per dish.
(9) Place the dishes at 37°C under 5% CO_2_.

#### Maintaining

(1) The day of passage, remove the medium and wash with 5 ml of PBS 1X.

NB: Pass the cells at 70% confluency.

(2) Add 2 ml of 0.25% trypsin solution and incubate 5 min at 37°C.
(3) Add 10 ml of OP9 medium and disaggregate the cells from the dish by pipetting them up and down.
(4) Transfer the cells to a 50 ml tube and centrifuge at 200 g for 5 min.
(5) Resuspend in 5 ml of OP9 medium and transfer 1 ml of cell solution into 10-cm dish previously coated with 0.1% gelatin and containing 9 ml of OP9 medium.

NB 1: Keep the split ratio at 1-to-5 for passaging the cells every 3–4 days. Passage the cells before they reach 80% confluency and do not keep them in culture for more than 5–7 weeks.

NB 2: DLL1 and DLL4 ligand expression by the OP9 cells can be monitored on a regular basis by flow cytometry to check that there is no loss of ligand expression due to culture (Supplementary Figure 1 and Supplementary Table 1)

### Generation of Lymphoid Progenitors

#### Preparation of OP9-DLL1 or OP9-DLL4 for Co-culture

(1) 24 h before starting the co-culture with OP9-DLL1 or OP9-DLL4 cells, coat 12-well plates with 0.1% gelatin for 30 min.

NB: OP9-DLL1 or OP9-DLL4 cells need to be at 70% confluency at day 8.

(2) Prepare the OP9-DLL1 or OP9-DLL4 cells as in step “Maintaining” (1–5).
(3) Resuspend the cells in 5 ml of OP9 medium. Count the number of cells and seed 3.5 × 10^4^ cells per 12-well plate.
(4) Be careful to homogenize the cells well and place them at 37°C under 5% CO_2_.

#### Dissociation of EBs to Recover HSPCs

(1) At day 9, collect one 6-well low binding plate of EBs with a 5 ml pipette and transfer them to a 15 ml tube. Rinse the well with 2 ml of α-MEM.

NB: One 6-well low binding plate in one 15 ml tube.

(2) Centrifuge for 5 min at 450 g.
(3) Remove the supernatant and resuspend in 1 ml of Accutase. Incubate 15 min at 37°C.
(4) Pipette up and down with 1 ml tips to break the EBs.

NB 1: This step requires about 5 min per tube to sufficiently break the EBs.

NB 2: If the EBs are not yet broken, it is possible to incubate an extra 5–10 min in 37°C.

(5) Add 5 ml of α-MEM and centrifuge 7 min at 450 g.
(6) Resuspend the HSPCs in 1 ml OP9 differentiation medium + hSCF (10 ng/ml), hFlt3L (5 ng/ml) and hIL-7 (5 ng/ml).

#### Co-culture With OP9-DLL1 or OP9-DLL4

(1) Remove the medium in OP9-DLL1 or OP9-DLL4 12-well plates and add 1 ml of OP9 differentiation medium + hSCF (10 ng/ml), hFlt3L (5 ng/ml) and hIL-7 (5 ng/ml).
(2) Add the 1 ml of HSPC solution into a 12-well plate of OP9-DLL1 or OP9-DLL4 and place the plate at 37°C under 5% CO_2_.

NB 1: One 6-well low binding of EBs in one 12-well plate coated with OP9-DLL1 or OP9-DLL4 cells.

NB 2: the cells are in a final volume of 2 ml OP9 differentiation medium plus hSCF (10 ng/ml), hFlt3L (5 ng/ml) and hIL-7 (5 ng/ml).

(3) Every 2 days, replace half of the medium. Remove 1 ml of medium from each well by leaning against the edge of the well with a 1 ml tip.
(4) Add 1 ml of OP9 differentiation medium + hSCF (20 ng/ml), hFlt3L (10 ng/ml) and hIL-7 (10 ng/ml).

NB: The final concentration of cytokines in 2 ml of OP9 differentiation medium is: hSCF (10 ng/ml), hFlt3L (5 ng/ml) and hIL-7 (5 ng/ml).

(5) Every 5 days, the cells are passed on new feeder layers of OP9-DLL1 or OP9-DLL4 cells.
(6) Prepare OP9-DLL1 or OP9-DLL4 cells as in step (1–4).
(7) To harvest the semi adherent hematopoietic cells, use the medium in the well and wash the cells very gently to keep the feeder layer intact.

NB 1: One 15 ml tube per well.

NB 2: If the feeder layer is broken in small clumps, just passage everything to a new layer.

NB 3: If the feeder layer comes off completely, try to recover the free cells but do not transfer the feeder layer mass.

(8) Transfer the cells in a 15 ml tube and rinse 2 times with 1 ml of α-MEM to recover all the cells.
(9) Centrifuge 10 min at 450 g.
(10) Resuspend the pellet in 1 ml of OP9 differentiation medium + hSCF (10 ng/ml), hFlt3L (5 ng/ml) and hIL-7 (5 ng/ml).
(11) Aspirate the OP9 medium from 12-well plates and add 1 ml onto OP9-DLL1 or OP9-DLL4 cells of OP9 differentiation medium with hSCF (10 ng/ml), hFlt3L (5 ng/ml) and hIL-7 (5 ng/ml).
(12) Add the cell solution into OP9-DLL1 or OP9-DLL4 cells.

NB: The final volume is 2 ml per well.

#### Flow Cytometry Analysis

(1) Resuspend the cells in 1 ml of FACS buffer and pass them through a 60 μm cell filter.
(2) Wash the cells with FACS buffer and centrifuge at 450 g for 7 min. Keep 1/3 of the cells form each condition and use them for unstained and isotype control conditions. Incubate the cells with anti-hCD34, anti-hCD43, anti-hCD45 and anti-hKDR antibodies in FACS buffer for 30 min at 4°C in the dark (Table 2).
(3) Wash the cells twice with FACS buffer and resuspend them in PBS 1X + DAPI.
(4) Analyze the cells via flow cytometry.

## Results

**FIGURE 1:**
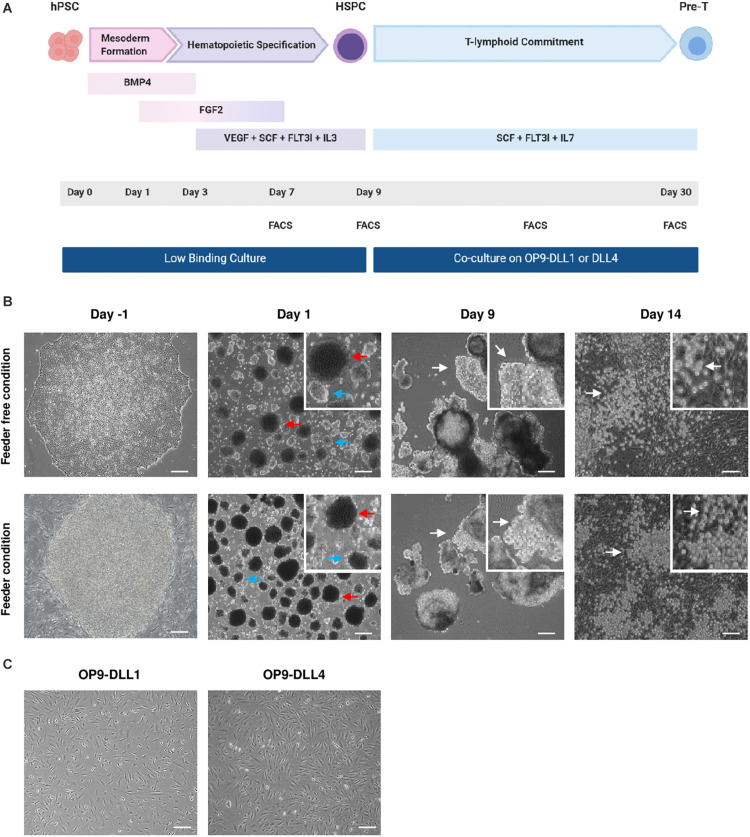
Generation and morphology of hematopoietic and lymphoid progenitors from hPSCs. **(A)** Schematic representation of the hHSPCs differentiation protocol. At D0, EBs are treated with BMP4 to induce mesoderm formation. At D1, the medium is supplemented with FGF2. Specification toward the hematopoietic lineage began on D3 with the withdrawal of BMP4 and the addition of VEGF, SCF, FLT3l, and IL-3 in the medium. The medium was changed at D1, D3, D5, and D7. After 9 days, the EBs were dissociated and the cells were co-cultured on OP9-DLL1 or DLL4 cells in a medium containing SCF, FLT3l, and IL-7 to induce T-lymphoid commitment. Every 5 days, cells were passed over new fresh OP9-DLL1 or DLL4 cells. Co-culture took about 20 days to produce lymphocyte progenitors. To follow the evolution of the differentiation, the cells were analyzed by flow cytometry on D7, D9, D19, and D30. **(B)** Representative photos of the cell during the culture. Prior to differentiation, hPSC are either on Matrigel (top) or on MEFs (bottom). At D1, there are two sizes of embryoid bodies; the biggest EBs indicated by the red arrow and the smallest indicated by the blue arrow. At D9, EBs have grown and formed clearer protrusions as indicated by the white arrows. After 5 days on OP9-DLL1 or DLL4 cells (D14), small rounded cells distributed in clusters are indicated by the white arrows. Scale bars, 200μm. Feeder-free condition: hES WA09, *n* = 8; Lon71.019, *n* = 5; MiPS209.003, *n* = 1, MiPS220.003, *n* = 2; T04.01A, *n* = 12; T04.01B, *n* = 4; T05.003, *n* = 3; T05.006, *n* = 4. Feeder condition: hES WA09, *n* = 11; hES WA01, *n* = 1; BJ1.B1, *n* = 1; LON71.019, *n* = 2; Lon80.B2, *n* = 1; MiPS203.B11, *n* = 1; T04.01A, *n* = 7. **(C)** Representative photos of OP9-DLL1/4 cells confluency before the co-culture with HSPCs. Scale bars, 200 μm.

Our objective was to design a protocol that would efficiently enable the differentiation of hPSC cultured in feeder-free conditions into hHSPCs (Figure 1A). In our hands, the differentiation of hPSCs into hHSPCs was more efficient with EBs in 3D culture, therefore we rapidly dropped the 2D hHSPCs induction protocol. Indeed, the 2D protocol was yielding less than 5% hHSPCs, was expensive and required sorting or magnetic separation of CD34 positive cells.

We evaluated the efficiency of EB formation and HSPC generation from hPSCs cultured on feeder or feeder-free conditions. Of note, we differentiated EBs without serum as it may contain inhibitory factors and it is difficult to troubleshoot the variability of serum batches. To monitor the efficiency of the differentiation, an informative readout was the morphology of EBs in both conditions. EBs were formed within 24 h after hPSC dissociation. The first 3 days (D0– D3), EBs grew slightly in size reaching 450–500 μm (Figure 1B). Between D3 and D5, EBs grew significantly and we observed protuberances coming out of the initially round shape of the EBs. Between D7 and D9, both EBs and protuberances grew significantly. Overall, the volume of EBs could triple in the course of the 9 days differentiation protocol (Figure 1B). Prior to any phenotypic or genotypic analysis, the morphology of the EBs, their growth and the appearance of protuberances ensured a good induction of the hematopoietic differentiation. Overall, EB formation from hPSCs cultured in feeder or feeder-free conditions behaved similarly.

**FIGURE 2:**
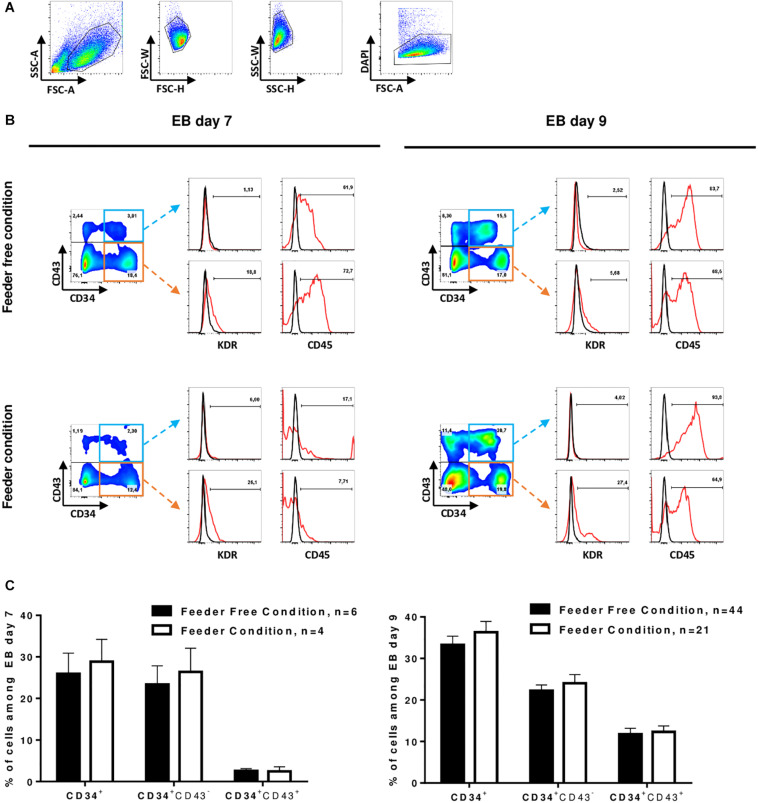
Induction of HSPCs. **(A)** Representative gating strategy for differentiated cells analysis by flow cytometry: differentiated cells were selected on morphology after exclusion of doublets and dead cells (DAPI). **(B)** Flow-cytometry analysis of differentiating cells at D7 (left) and D9 (right) in EB culture from hPSC in feeder-free condition (top) and in feeder condition (bottom). Representative dot plot of CD34 and CD43 co-staining on living cells is shown on the left. The expression of KDR and CD45 in CD34^+^CD43^+^ (top) or CD34^+^CD43^–^ (bottom) cells is shown on the right. Red line represents cells stained with a fluorescent antibody and black line represents unstained cells. **(C)** Percent of total cells from EBs day 7 (left) and day 9 (right) expressing the markers, Mean ± SEM are represented.

At D7 and D9, we monitored the hematopoietic differentiation by flow cytometry to show the acquisition of key markers of the hematopoietic lineage. We analyzed the expression of 4 markers to identify hematopoietic stem cells: CD34 as the major human hematopoietic stem cells (hHSCs) marker (Berenson et al., 1988), CD43 as an early marker of hematopoiesis (Vodyanik et al., 2006), CD45 as the most common marker for hematopoietic cells (Vodyanik et al., 2006) and KDR as an endothelial cell marker also commonly used to identify hemangioblasts (Kennedy et al., 2012; Sturgeon et al., 2014). At D7, CD34 marker expression averaged 27 ± 12.1% and at D9, this expression reached 37 ± 13.7% of the total culture in both feeder and feeder-free culture conditions (tested on 12 cell lines). Between D7 and D9, we observed the acquisition of the CD43 marker in CD34^+^ cells. There was an average of 3 and 13% CD34^+^CD43^+^ cells at D7 and D9, respectively (Figures 2A–C). However, we cannot exclude that part of CD43^+^ cells from D7 may proliferate and contribute to this increase of CD43^+^ cells between D7 and D9. Of note, weak expression of KDR, an endothelium marker (Sturgeon et al., 2014), was detected at D7 only in cells cultured on feeder cells prior to differentiation. At D7, in the feeder condition, CD34^+^ cells had little expression of the CD45 marker, while in contrast, in the feeder-free condition, the CD34^+^CD43^–^ cells already expressed CD45 (Figure 2B). In the feeder condition, the expression of KDR decreased while the expression of CD45 was increased in CD34^+^CD43^+^ cells between D7 and D9. Feeder-free cultured hPSCs therefore seem to differentiate slightly faster than feeder culture ones. After 9 days of differentiation of hPSCs into EBs, the differentiated cells had identical phenotypes regardless of whether they came from feeder or feeder-free systems: there was an average of 37 ± 13.7% CD34^+^ cells in the total population, all of them expressing CD45 (Figures 2B,C).

**FIGURE 3:**
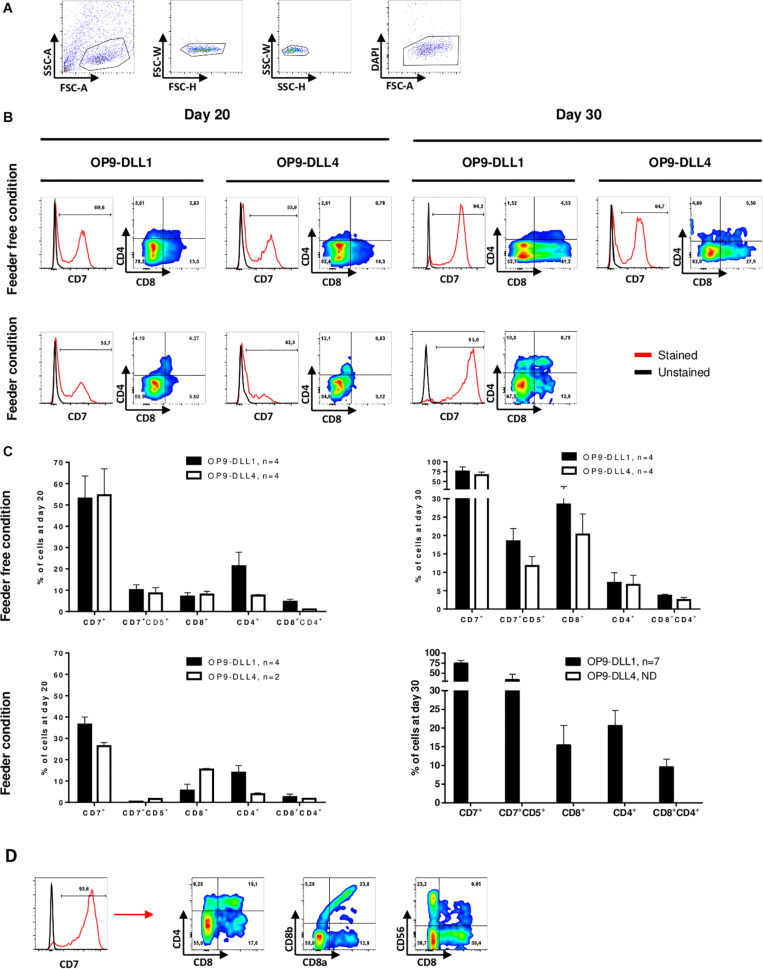
T cell progenitors’ differentiation is neither dependent on hPSCs culture conditions nor lymphopoietic induction by OP9-DLL1 or DLL4. **(A)** Representative gating strategy for differentiated cells analysis by flow cytometry: differentiated cells were selected on morphology after exclusion of doublets and dead cells (DAPI). **(B)** Representative histogram of CD7 expression in living cells (excluding OP9) on OP9-DLL1 (left) or OP9-DLL4 (right) from feeder-free condition (top) and feeder condition (bottom) at day 20 and day 30. Red line represents cells stained with a fluorescent antibody and black line represents unstained cells. The expression of CD8 and CD4 among CD7^+^ cells is shown in dot plot. **(C)** Percent of total living cells (excluding OP9) at day 20 and day 30 in OP9-DLL1 or in OP9-DLL4 expression the markers, Mean ± SEM are represented. **(D)** Representative histogram of CD7 expression in living cells (excluding OP9) on OP9-DLL1 from feeder condition at day 35. Red line represents cells stained with a fluorescent antibody and black line represents unstained cells. The expression of CD8α, CD8β, CD4, and CD56 among CD7^+^ cells is shown in dot plot.

After establishing robust hematopoietic induction of hPSC cultured in feeder-free conditions, we sought to optimize lymphopoiesis induction. To do so, we assessed co-culture of hHSPCs differentiated from hPSCs for 9 days with the OP9 line of mouse bone marrow stromal cells constitutively expressing DLL1 or DLL4. Both DLL1 and DLL4 have been used in lymphopoiesis protocols with human PSCs (La Motte-Mohs et al., 2005; Mohtashami et al., 2013; Nishimura et al., 2013; Themeli et al., 2013), but few studies have compared the two cell types. EBs were dissociated at day 9 using accutase and the cell suspension, consisting of individualized cells and some small clumps, was seeded on top of OP9-DLL1 or OP9-DLL4 cells (Figure 1C). Between D10 and D13, some individualized cells were detached from the small clumps. By day 14, we generally observed small rounded cells, often distributed in clusters (Figure 1B). We monitored progression toward the T lymphocyte fate by flow cytometry analysis using the following surface markers: CD7 as an early marker of the T-cell lineage (Lobach et al., 1985), CD8 and CD4 as commonly used to identify the two main types of T cells (Miceli and Parnes, 1991). At least 40% of cells co-cultured on OP9-DLL1 or OP9-DLL4 were CD7 positive by day 20 (Figures 3A–C). This was not modulated by OP9-DLL1 or OP9-DLL4 co-culture. We observed a trend for the feeder condition to be less potent at inducing CD7 expression than feeder-free culture of hPSCs prior to differentiation (36.4 ± 7.2% vs. 53.8 ± 21.4%, respectively). At day 20, about 5% of CD7^+^ cells expressed the CD4 marker whereas a higher proportion expressed the CD8 marker (15%) and only a small proportion (1–2%) were double positive CD4^+^CD8^+^ in feeder-free condition (Figure 2C). At least 70% of the cells were CD7^+^ by day 30 and 5–10% of cells were CD4^+^CD8^+^ (Figures 2B,C). These CD7^+^CD4^+^CD8^+^ cells were progenitors of the T lineage. The efficiency of differentiation at day 30 was not affected either by the OP9-DLL1 or OP9-DLL4, or by the feeder/feeder-free culture of hPSCs prior to differentiation. Moreover, we did not observe any influence of the source of cells: hESCs or hiPSC reprogramed from fibroblast, lymphocytes (10 hiPSC and 2 hESC lines were used). To further demonstrate that these progenitors are bona fide DP T cells, we further differentiated them into CD8α^+^CD8β^+^ cells and CD4^+^CD8^–^ cells (SP) T cells in the feeder condition on OP9-DLL1 (Figure 3D). In addition, we show the presence of CD56^+^ cells at day 35. Digital gene RNA sequencing (DGE-RNAseq) data from D25 cells compared to CD8^+^CD4^+^ thymus cells demonstrated that T cells on OP9-DLL1 and DLL4 feeder free and OP9-DLL1 feeder cells expressed similar levels of lymphoid lineage genes and cytotoxicity mediators than CD8^+^CD4^+^ cells from thymus (Supplementary Figure 2). Indeed, cells at day 25 express key genes for lymphopoiesis such as GATA3, IL7R, BCL11b, CD25, as well as PTCRA, a gene involved in the formation of the pre-TCR complex and all chains of CD3. In addition, we observed the expression of cytotoxic mediators such as GNLY, GZMB, TNFSF10, and TYROBP. This altogether demonstrates that our hHSPCs derived from EB differentiation from hPSCs were able to differentiate to T cell progenitors.

**FIGURE 4:**
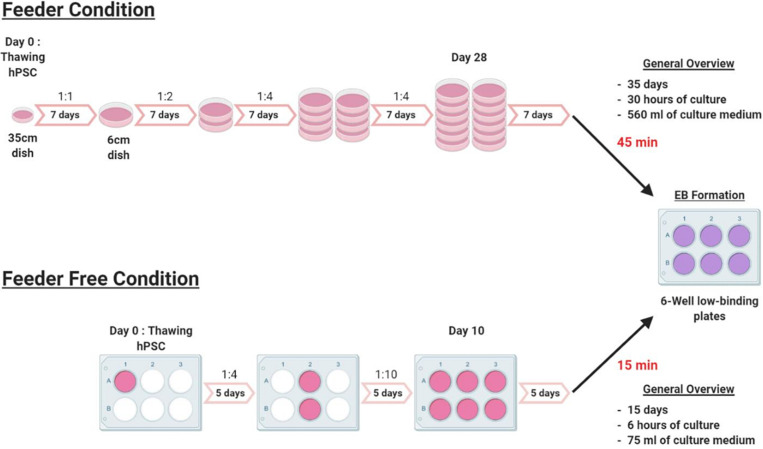
Experimental outline. Schematic representation of the experimental outline in feeder and feeder-free culture conditions, from thawing to EB formation. The feeder condition takes 35 days of culture with a weekly passage at a ratio that varies from 1:1 to 1:4. EBs formation takes about 45 min from 12 × 6 cm dishes to 6-wells low binding plates of EBs. In feeder-free culture condition, the culture time is reduced to 15 days with a passage every 5 days at a ratio that varies from 1:4 to 1:10. The time for EBs formation is also significantly reduced from 45 min to only 15 min.

To assess the value of our protocol, we compared the time necessary for each setup (Figure 4). Expansion of hPSCs in feeder-free culture conditions is 3 weeks faster than in feeder culture conditions, prior to differentiation. Indeed, with feeders, from thawing to EB formation required about 35 days to have 12 dishes ready. In the feeder-free culture conditions, it required only 10–15 days. Also, in feeder-free culture conditions it took about 15 min to dispense cells in a 6-wells plate for the passages and the EBs formation, whereas, in the feeder condition it took 45 min. Our protocol to differentiate hPSCs cultured in feeder-free conditions will thus tremendously help laboratories and groups working in this field since feeder-free culture of hPSCs requires 70% reduced hands-on time than feeder cultures. The full protocol was tested and validated for hES WA09, T04-01A, T04-01B, T05-006, MiPS209-003, MiPS220-003 and we did not observe qualitative and quantitative differences between cell lines.

## Conclusion/Discussion

The goal of our study was to propose a rapid and efficient protocol to drive hPSCs toward the T lymphocyte fate. To do so, we assessed the effect of the culture conditions on the EB formation and hematopoietic induction. Feeder-free conditions were required to be GMP compatible and interestingly, all aspects of the differentiation setup were improved using feeder-free conditions: hPSCs amplification, passaging time and EB formation. Those technical improvements did not impair efficiency. The yield was similar in feeder-free compared to feeder conditions, regardless of the source of hPSC lines (hESC: *n* = 2, fibroblast-hiPSC: *n* = 6, T-cell hiPSC: *n* = 4).

Several studies reported the importance of EB size for the differentiation of hPSCs (Bauwens et al., 2008). In our study, the EB formation was done by hand scrape. With this technique, we mainly obtained two sizes of EBs, small ones around 100 μm diameter and larger ones around 200 μm diameter. In our hands, the size of EBs did not impact the differentiation efficiency. Therefore, we could keep the protocol simple and avoid using single PSC suspension and EB formation through culture in patterned microwells, such as AggreWells (Ng et al., 2005; Antonchuk, 2013). A critical factor indicating successful differentiation was the morphological aspect of EBs.

Using either feeder or feeder-free conditions, we observed two distinct populations, CD34^+^CD43^+^CD45^+^KDR^–^ cells, which were hematopoietic progenitors, and CD34^+^CD43^–^CD45^+^KDR^–^ cells, which were in a less advanced state of hematopoiesis differentiation. We chose not to sort CD34^+^CD43^+^ cells for seeding on OP9-DLL1/4. Indeed, our experience has shown that sorting CD34^+^CD43^+^ cells with FACS Aria is traumatic for the cells and results in significant mortality of these progenitors. In our hands, seeding the full disaggregated EB on OP9-DLL1/4 led to less progenitor mortality. We did not observe adverse effect of seeding all cells on the differentiation outcome.

We observe two cell populations that are likely to become T lymphocytes: CD34^+^CD43^+^CD45^+^KDR^–^ and CD34^+^CD43^–^CD45^+^KDR^–^ subsets. The CD34^+^CD43^+^CD45^+^KDR^–^ will be directed toward the T lymphocyte fate by the contact with DLL1 or DLL4 ligands that activate the essential Notch signaling pathway (García-León et al., 2018) and by the cocktail of cytokines supplementing the culture medium (SCF, FLT3l and IL-7). We noticed that CD34^+^CD43^–^CD45^+^KDR^–^ cells still expressed high levels of endothelial markers such as PECAM1, VE-Cadherin, or YAP1 unlike CD34^+^CD43^–^CD45^+^KDR^–^ cells. Therefore, CD34^+^CD43^–^CD45^+^KDR^–^ cells might not have accomplished endothelium-hematopoietic transition. Nevertheless, Notch signaling could trigger CD34^+^CD43^–^CD45^+^KDR^–^ cells to transit to a progenitor state able to differentiate toward the T lineage (Kennedy et al., 2012; Sturgeon et al., 2014).

Two landmark studies compared DLL1 and DLL4 potency to induce T lymphocyte differentiation (Besseyrias et al., 2007; Mohtashami et al., 2010). They demonstrated that in human HCSs, DLL4 induced a Notch pathway response closer to the physiological conditions than DLL1 (Mohtashami et al., 2010). They also showed that DLL1 and DLL4 allowed differentiation toward the T lineage in a similar manner up to the pre-T CD25^+^ stage but the maturation afterward was favored by DLL4 than by DLL1 (Besseyrias et al., 2007).

In our study, we did not observe any significant differences in the induction of T lymphocyte progenitors from hPSC-derived hHSPCs in presence of DLL1 or DLL4. We concluded that DLL1 and DLL4 might have the same potency to drive early lymphopoiesis. In both cases, after 20 days of co-culture, we obtained precursors of the T lineage: CD7^+^CD4^+^CD8^+^, CD7^+^CD4^+^CD8^–^ and CD7^+^CD4^–^CD8^+^ cells which did not express the CD3 and TCRαβ markers.

We showed by flow cytometry at day 35 and RNA-DGE sequencing analysis of differentiated cells at day 25, that these progenitors are bona fide DP T cells since they expressed key genes for lymphopoiesis, cytotoxic mediators and were able to further differentiated into CD8α^+^CD8α^+^ cells and CD4^+^CD8^–^ cells (SP) T cells. In addition, we detected the expression of 20–30% CD56 positive cells, likely natural killer lymphocytes, in line with previous reports (Nishimura et al., 2013; Themeli et al., 2013).

Advances in immune cell engineering are opening up new perspectives for the generation of custom synthetic T cells from hiPSCs. TCR acquisition by rearrangement during *in vitro* differentiation will no longer be a problem since antigen specificity can be given to hiPSCs and their T cell derivatives by means of a transgenic TCR (Minagawa et al., 2018). Themeli et al. have demonstrated the feasibility of generating functional *in vitro* and *in vivo* chimeric antigen receptor (CAR)-T cells by engineering T-iPSCs with a CAR before differentiation (Themeli et al., 2013). The use of CARs provides T-iPSC-derived T cells customizable antigen specificity in a HLA-independent manner. Importantly, second and third generation CARs provide additional costimulatory signals and improve *in vivo* T cell activation, expansion and persistence. In principle, like CAR, T-iPSCs can also be genetically armed with cytokines, receptors and other regulatory molecules to confer optimal immunotherapeutic properties such as increased proliferation, and allogeneic HLA molecules can be knocked-out to generate stealth T cells that can evade detection and elimination by the host immune system (Nianias and Themeli, 2019).

Generation of T cell progenitors *in vitro* offers the opportunity to study lymphopoiesis. T cells are circulating in the blood which makes them easy to study. However, it remains extremely difficult to study the genesis of T cells in human. Recent strategies consist of performing single cell RNA sequencing from thymus and reconstituting fate trajectories (Park et al., 2020). This requires access to human thymuses and does not allow to functionally test hypotheses. Thus, *in vitro* generation of T cells would allow unprecedented investigation of lymphopoiesis.

To conclude, we established a robust, easy to implement and GMP friendly protocol for the generation of HSPCs, using feeder-free hPSC culture conditions. This protocol yielded over 13% of CD34^+^CD43^+^CD45^+^KDR^–^ hHSPCs. We also established a protocol for T cell progenitors that would require further developments to be GMP-compatible, in particular culture conditions that would alleviate the need for OP9 cells. Our investigations showed that there were no major differences in the use of OP9-DDL1 or DLL4 cells for induction of lymphopoiesis. Like other groups before us (Kyttälä et al., 2016; Nishizawa et al., 2016), the only slight difference we observed was the variation between the different hPSC lines, likely due to the genetic background of each of the cell lines. Altogether, our protocol enables a much-needed approach to understand the development of the immune system in human. This opens new opportunities for drug screening, disease modeling and could provide an unlimited number of cells for regenerative medicine and cell therapy.

## Data Availability Statement

All datasets generated for this study are included in the article/Supplementary Material.

## Author Contributions

CG and LD: conceptualization, supervision, project administration, and funding acquisition. LF, AG, MT, CG, and LD: methodology and writing – review and editing. LF, AG, CG, and LD: validation. LF, AG, and CS: formal analysis. LF and AG: investigation. OB and XS: contributing essential reagent. LF: writing – original draft. All authors contributed to the article and approved the submitted version.

## Conflict of Interest

The authors declare that the research was conducted in the absence of any commercial or financial relationships that could be construed as a potential conflict of interest.
