# Supplementary material for: Rapid and Reproducible Differentiation of Hematopoietic and T Cell Progenitors From Pluripotent Stem Cells
Source: Front Cell Dev Biol. 2020 Oct 20;8:577464. doi: 10.3389/fcell.2020.577464 (PMC7606846; doi:10.3389/fcell.2020.577464)
Supplement: Supplementary file 1 [file Data_Sheet_1.docx]

**
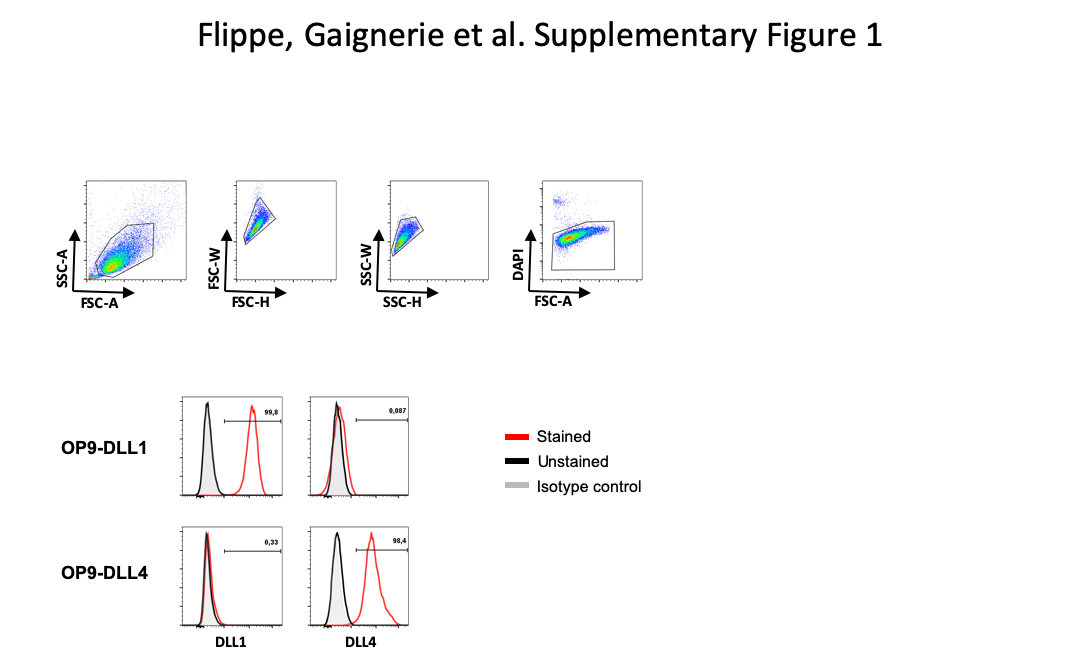
**

**Supplementary Figure 1 :** **DLL1 and DLL4 expression by OP9 cells**

Flow cytometric analysis is shown for cell surface expression of DLL1 and DLL4 on OP9-DLL1 and OP9-DLL4. Red line represents cells stained with a fluorescent antibody, black line represents unstained cells and the isotype control in grey.


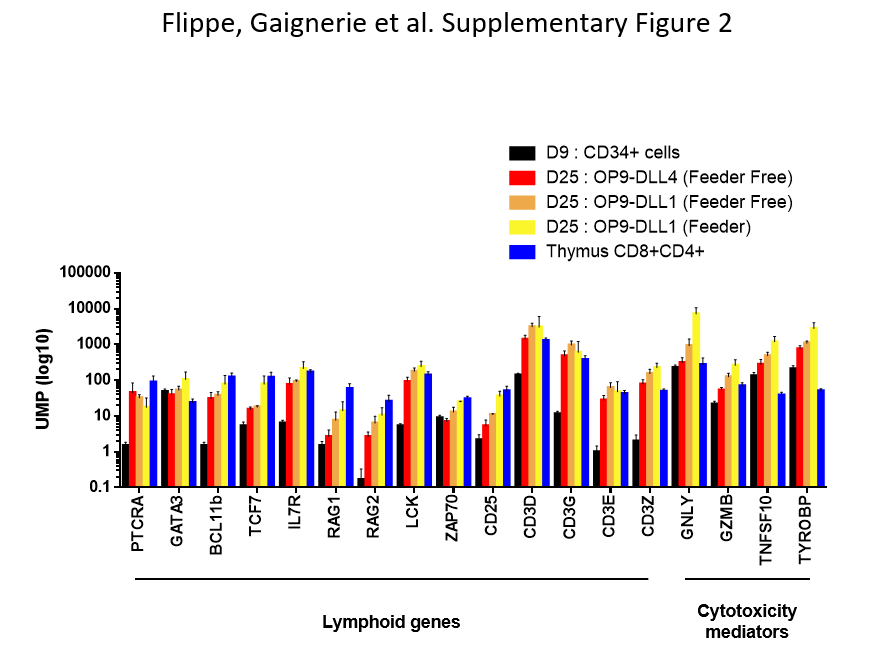


**Supplementary Figure 2 :** **Induction of lymphoid lineage genes by differentiated cells**

D25 cells were analyzed by DGE RNAseq and compare to CD34^+^ cells from day 9 and CD8^+^CD4^+^ thymus cells. Selected lymphoid lineage genes and cytotoxicity mediators were plotted. Mean +/- SEM are represented.
