# Supplementary material for: Rapid and Reproducible Differentiation of Hematopoietic and T Cell Progenitors From Pluripotent Stem Cells
Source: Front Cell Dev Biol. 2020 Oct 20;8:577464. doi: 10.3389/fcell.2020.577464 (PMC7606846; doi:10.3389/fcell.2020.577464)
Supplement: Supplementary file 2 [file Data_Sheet_2.docx]

**Supplementary method**

***Thymus cells sorting***

Human thymi are recovered after childhood heart surgery at Nantes University Hospital. Thymus were shredded using needles to extract cells; cells were sorted by BD FACSAria (BDBiosciences) by gating on DAPI^-^CD3^+^CD8^+^CD4^+^.

***DGE-RNA sequencing***

Total RNA was extracted using Rneasy columns and DNAse-treated using RNase-free DNase (Qiagen). 3′digital gene expression (3′DGE) RNA-sequencing protocol was performed according to25. Briefly, the libraries were prepared from 10 ng of total RNA. The mRNA poly(A) tail were tagged with universal adapters, well-specific barcodes and unique molecular identifiers (UMIs) during template-switching reverse transcriptase. Barcoded cDNAs from multiple samples were then pooled, amplified and tagmented using a transposon-fragmentation approach which enriches for 3′ends of cDNA. A library of 350–800 bp was run on an Illumina NovaSeq 6000 (NovaSeq 6000 SP Reagent Kit 100 cycles (ref #20027464)). Read pairs used for analysis matched the following criteria: all sixteen bases of the first read had quality scores of at least 10 and the first six bases correspond exactly to a designed well-specific barcode. The second reads were aligned to RefSeq human mRNA sequences (hg19) using bwa version 0.7.4 4 with non-default parameter “-l 24”. Reads mapping to several positions into the genome were filtered out from the analysis. Digital gene expression (DGE) profiles were generated by counting for each sample, the number of unique UMIs associated with each RefSeq genes. DGE-sequenced samples were acquired from three sequencing runs. All samples with more than 200 000 assigned reads were retained for further analysis. DESeq 2 was used to normalize expression with the DESeq function. Normalized counts were transformed with vst (variance stabilized transformation) function from DESeq library. Genes expressions were presented in UPM (UMI per million) in histograms. ENA Study accession number is PRJEB40374.

***Mitotically-inactivated MEFs***

Mouse embryonic fibroblast (MEF) must be healthy and actively dividing prior to inactivation and their subsequent use as a feeder layer for PSCs. In brief, MEFs are thawed in 15-cm dish coated with gelatin and containing MEF medium (DMEM Glutamax high glucose supplemented with 10% fetal bovin serum, 1% of sodium pyruvate, 1% of nonessential amino acids and 0.5% of Penicillin-Streptomycin). When the cells are confluent, they are passed with trypsin treatment and seeded at a density of 10*10^6^ per 15-cm dish. After reconstitution in MEF medium, Mitomycin C (Sigma) is added up to 10μg/mL. Mitomycin C is incubated with the cells for 2-3 hours. Then, cells are washed to remove all traces of mitomycin and freeze for later use.
