# Supplementary material for: Rapid and Reproducible Differentiation of Hematopoietic and T Cell Progenitors From Pluripotent Stem Cells
Source: Front Cell Dev Biol. 2020 Oct 20;8:577464. doi: 10.3389/fcell.2020.577464 (PMC7606846; doi:10.3389/fcell.2020.577464)
Supplement: Supplementary file 3 [file Data_Sheet_3.docx]

**Supplementary Table 1 :** Antibodies used for analysis of OP9-DLL1 and OP9-DLL4

| Antigen | Clone | Conjugate | Supplier | Catalog # | Dilution |
| --- | --- | --- | --- | --- | --- |
| Delta-Like Protein 1 | 30B11.1 | BV605 | BD Biosciences | 745173 | 1/30 |
| Delta-Like Protein 4 | 9A1.5 | BUV737 | BD Biosciences | 748394 | 1/30 |
| Isotype control  Rat IgG2a,k | 2D1 | BV605 | BD Biosciences | R35-95 | 1/30 |
| Isotype control  Rat IgG1 | R3-34 | BUV737 | BD Biosciences | 612770 | 1/30 |
